# Dihydroartemisinin inhibits catabolism in rat chondrocytes by activating autophagy via inhibition of the NF-κB pathway

**DOI:** 10.1038/srep38979

**Published:** 2016-12-12

**Authors:** Li-Bo Jiang, De-Hua Meng, Soo-Min Lee, Shu-Hao Liu, Qin-Tong Xu, Yang Wang, Jian Zhang

**Affiliations:** 1 Department of Orthopedic Surgery, Zhongshan Hospital, Fudan University, Shanghai, China

## Abstract

Osteoarthritis is a disease with inflammatory and catabolic imbalance in cartilage. Dihydroartemisinin (DHA), a natural and safe anti-malarial agent, has been reported to inhibit inflammation, but its effects on chondrocytes have yet to be elucidated. We investigated the effects of DHA on catabolism in chondrocytes. Viability of SD rats chondrocytes was analyzed. Autophagy levels were determined via expression of autophagic markers LC3 and ATG5, GFP-LC3 analysis, acridine orange staining, and electron microscopy. ATG5 siRNA induced autophagic inhibition. Catabolic gene and chemokine expression was evaluated using qPCR. The NF-κB inhibitor SM7368 and p65 over-expression were used to analyze the role of NF-κB pathway in autophagic activation. A concentration of 1 μM DHA without cytotoxicity increased LC3-II and ATG5 levels as well as autophagosomal numbers in chondrocytes. DHA inhibited TNF-α-induced expression of MMP-3 and -9, ADAMTS5, CCL-2 and -5, and CXCL1, which was reversed by autophagic inhibition. TNF-α-stimulated nuclear translocation and degradation of the p65 and IκBα proteins, respectively, were attenuated in DHA-treated chondrocytes. NF-κB inhibition activated autophagy in TNF-α-treated chondrocytes, but p65 over-expression reduced the autophagic response to DHA. These results indicate that DHA might suppress the levels of catabolic and inflammatory factors in chondrocytes by promoting autophagy via NF-κB pathway inhibition.

The most common joint disease, osteoarthritis (OA), leads to absenteeism at work due to disability associated with joint pain and dysfunction^1^. Risk factors including obesity, aging, trauma, and lipid metabolism are etiological factors in OA. However, the definitive mechanism underlying the progression of OA remains controversial^2^. During the course of OA, low-grade inflammation with increased expression of proinflammatory cytokines (including TNF-α and IL-1β) in articular cartilage and synovium contribute to an increase in matrix metalloproteinases (MMPs) and chemokines in the extracellular matrix (ECM) and chondrocytes, resulting in cartilage degradation and erosion^3^. MMPs consist of disintegrins and metalloproteinases with thrombospondin motifs (ADAMTSs). Therefore, disruption of homeostasis in cartilage metabolism and a shift of cellular phenotype determine joint degeneration. However, reliable and effective drugs that could attenuate inflammation and maintain the balance of chondrocyte metabolism have yet to be developed or discovered.

Artemisinin (ART), a well-known and efficacious malarial drug, is extracted from *Artemisia annua*^4^. Dihydroartemisinin (DHA), a semisynthetic derivative of ART and a novel anti-malarial agent, has fewer side effects than ART^5^. The biological activity of DHA in addition to its anti-malarial role suggests that it inhibits growth of cancer cells, suppresses estrogen deficiency-induced osteoporosis and osteoblast remodeling, and inhibits tumor angiogenesis^6^,^7^. The inhibitory role of DHA on inflammation- and catabolism-associated genes has also been reported^8^,^9^. Its effect on chondrocytes, however, remains unclear.

Autophagy, is an intrinsic self-protective mechanism, which degrades excessive proteins and organelles by fusing with lysosomes in cells under stress^10^. Currently, researchers suggest that autophagy is protective in OA, although it has a dual effect on cell viability and function. Activation of autophagy in chondrocytes might attenuate OA progression via intra-articular injection or intra-peritoneal injection of rapamycin^11^,^12^. Inhibition of autophagy by 3-methyladenine (3-MA) or RNAi, however, increased apoptosis in chondrocytes^13^. Moreover, both IL1-β-induced MMP1-3 and ADAMTS5 were decreased by autophagic activation after rapamycin treatment^14^. Our previous research also indicated that autophagy might mediate the inhibitory role of adipose-derived stem cells on catabolism in chondrocytes^15^. Previous findings indicated that autophagy may be associated with chondrocyte homeostasis and cartilage metabolism. Therefore, we hypothesized that autophagy might be involved in DHA-mediated effects in chondrocytes. In the present study, ATG5 and LC3-II were used to detect the autophagy. During the formation of autophagosomes and autolysosomes, several autophagy-related genes (ATGs) are required^16^. Microtubule-associated protein 1 light chain 3 (also known as Atg8, LC3) and ATG5 are required for autophagosome formation and maturation^17^. LC3-I is transformed into LC3-II by binding with phosphatidylethanolamine (PE) and dispersion in the outer and inner membranes of the autophagosomes during the formation of the phagophore and autophagosomes^17^. Therefore, LC3-II and ATG5 are possible autophagic markers in mammals.

An increasing number of studies have reported that DHA inhibits nuclear translocation of the nuclear factor-kappa B (NF-κB)-associated pathway^9^. NF-κB is a key transcription factor regulating inflammation and cell proliferation^18^. Upon activation, IκBα is degraded, accompanied by nuclear translocation of NF-κB and downstream gene transcription. Recently, many researchers have found that inhibition of the NF-κB pathway triggers autophagy in both cancer and nuclear pulposus cells^19^,^20^. In the present study, we explored DHA effects on TNF-α-induced catabolism in chondrocytes, as well as the role of NF-κB pathway in autophagy.

## Results

### DHA effects on chondrocyte viability

**Figure 1:**
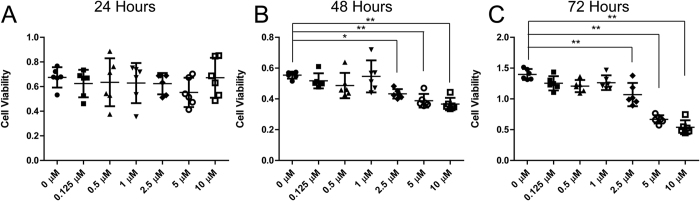
Cell viability under dihydroartemisinin (DHA) stimulation. (**A**,**B**,**C**) Chondrocytes treated with 0‒10 μM DHA for 24‒72 hours were analyzed by the CCK-8 test. Data represent mean ± 95% confidence interval (CI)*P < 0.05, **P < 0.01, n = 6.

Cell viability was estimated by the CCK-8 test. Twenty-four hours after treatment with DHA, the optical density value representing cell viabilities was similar among the groups treated with 0‒10 μM DHA (Fig. 1A). However, after 48 and 72 h treatments, 2.5 μM DHA significantly inhibited cell viability, and concentrations of 1 μM DHA or less had no harmful effects on cellular viability (Fig. 1B,C). Therefore, we used the 1 μM DHA dose for 24 hours in the subsequent experiments to prevent cytotoxicity.

### DHA activates autophagy in TNF-α-treated chondrocytes

**Figure 2:**
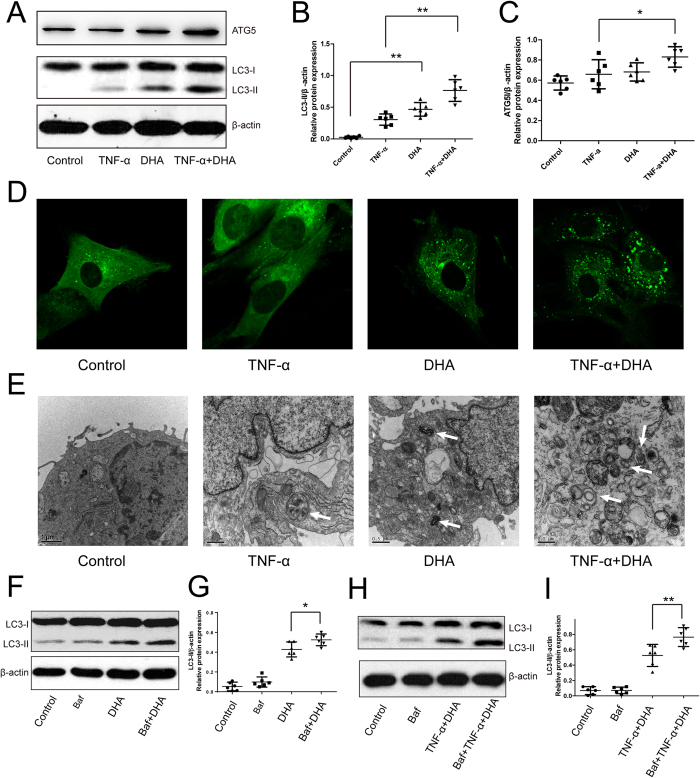
Effects of DHA on autophagy in TNF-α-treated chondrocytes for 24 hours. Representative images are shown. (**A**) Chondrocytes treated with TNF-α and/or DHA for 24 hours, and ATG5 and LC3-II expressions were analyzed by Western blotting. (**B**,**C**) The optical density of LC3-II/β-actin and ATG5/β-actin was analyzed. Data represent mean ± 95% CI, *P < 0.05, **P < 0.01, n = 6. (**D**) GFP-LC3 assay was used to detect the number of autophagosomes in chondrocytes. (**E**) The ultra-structures in chondrocytes were examined by TEM. The white arrow indicates autophagosome and autolysosomes in the cytoplasm. (**F**,**G**) Western blotting of LC3 proteins following treatment with DHA (1 μM) and/or bafilomycin A1 (Baf; 0.1 μM) for 24 hours, followed by determination of the optical density of LC3-II/β-actin. The values represent mean ± 95% CI, *P < 0.05, n = 6. (**H**,**I**) Treatment with DHA combined with TNF-α and/or Baf (0.1 μM) for 24 hours, was followed by Western blotting of LC3 levels and analysis of the optical density of LC3-II/β-actin. The values represent mean ± 95% CI, **P < 0.01.

LC3 is a classic marker of autophagic levels. Western blotting of LC3 and GFP-LC3 assays, therefore, were used to demonstrate the stimulatory effects of DHA on autophagy. One micromolar DHA significantly enhanced LC3-II expression in the TNF-α-treated chondrocytes for 24 hours (Fig. 2A,B). Interestingly, we found that LC3-II expression was increased in chondrocytes treated with DHA alone at 1 μM concentration, which was lower than that in cells co-treated with DHA and TNF-α, indicating that inflammation enhances stimulatory effect of DHA on autophagy (Fig. 2A,B). Consistent with the LC3-II level, the ATG5 levels were significantly increased following co-treatment with DHA and TNF-α, compared with TNF-α alone for 24 hours (Fig. 2A,C). GFP–LC3 is also widely used to monitor accumulation of the cytoplasmic puncture LC3 protein and autophagosomes^21^. Twenty-four hours after GFP–LC3 adenovirus transfection, the success and efficiency of transfection in chondrocytes was validated by fluorescence microscopy. The number of green GFP–LC3 dots in DHA-treated chondrocytes under inflammatory conditions induced by TNF-α was higher than that in TNF-α-treated chondrocytes (Fig. 2D), suggesting accumulation of autophagosomes in the chondrocytes. TEM is the gold standard by which autophagy activation is checked^21^. Autophagosomes and autolysosomes with double membranes appeared in the TNF-α- and DHA-treated chondrocytes for 24 hours (Fig. 2E). DHA significantly enhanced the number of autophagosomes in TNF-α-treated chondrocytes compared with DHA-treated cells (Fig. 2E).

We further investigated the autophagic flux via addition of bafilomycin A1 to inhibit the fusion of lysosomes with autophagosomes during the late phase of autophagy, leading to LC3-II accumulation in the cytoplasm. Bafilomycin A1 significantly elevated the LC3-II/β-actin level in DHA-treated chondrocytes, suggesting increased autophagic flux by DHA (Fig. 2F,G). Furthermore, the stimulatory role of DHA combined with TNF-α on autophagic flux was also demonstrated by the increase in LC3-II/β-actin level following the addition of bafilmycin A1 (Fig. 2H,I).

### Autophagy mediates the inhibitory effect of DHA on catabolic genes under inflammatory conditions

**Figure 3:**
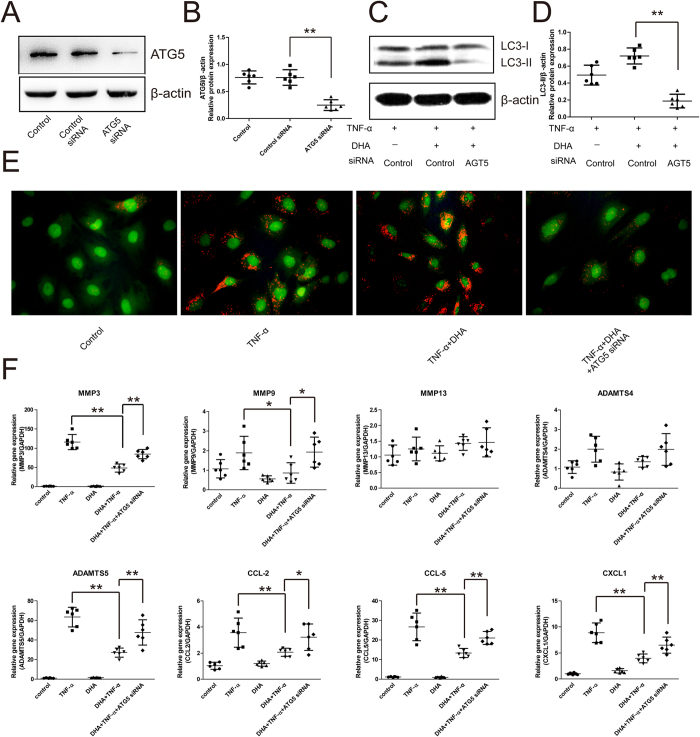
The involvement of autophagy in DHA inhibition of catabolic genes in chondrocytes under inflammatory conditions for 24 hours. Representative images are shown. (**A**,**B**) The expression of ATG5 in chondrocytes 48 hours after ATG5-siRNA transfection was analyzed by Western blotting, and the optical density of ATG5/β-actin was analyzed. Data represent mean ± 95% CI, *P < 0.05, **P < 0.01, n = 6. (**C**,**D**) Western blotting analysis of LC3 levels in chondrocytes after ATG5 silence. Data represent mean ± 95% CI, *P < 0.05, **P < 0.01, n = 6. (**E**) AO staining for acidic vesicular organelles in chondrocytes 48 hours after transfection with ATG5 siRNA. F. 48 hours after ATG5 silencing, mRNA levels of MMP-3 and -13, ADAMTS-4 and -5, CCL-2 and -5, and CXCL1 in TNF-α- and/or DHA-treated chondrocytes were analyzed by real-time PCR. Data represent as mean ± 95% CI, *P < 0.05, **P < 0.01, n = 6.

In order to investigate the involvement of autophagy in DHA inhibition of TNF-α- induced catabolic genes, RNAi technology against ATG5 (regarded as a key protein of autophagosome formation) was used to inhibit autophagic levels. After ATG5 siRNA transfection, the decrease in ATG5 protein levels in chondrocytes was examined using Western blotting (Fig. 3A,B). Under TNF-α-induced inflammatory conditions, ATG5 silencing reversed the increase in DHA-induced LC3-II levels in chondrocytes (Fig. 3C,D). In accordance with the results obtained from the LC3-II level analysis, ATG5-silenced chondrocytes treated with DHA and TNF-α for 24 hours showed fewer acidic vesicular organelles. They were detected as red-stained dots under fluorescence microscopy when compared with the normal chondrocytes (Fig. 3E), indicating fewer autophagosomes in the ATG5-silenced chondrocytes. The mRNA levels of catabolic genes, including MMP-3, -9, and -13, ADAMTS4 and -5, CCL-2 and -5, and CXCL1 in chondrocytes were investigated by real-time PCR. Interestingly, DHA significantly attenuated the TNF-α-induced increases in MMP-3 and -9, ADAMTS5, CCL-2 and -5, and CXCL1 (Fig. 3F). However, DHA inhibition of MMP-3, ADAMTS5, CCL-2 and -5, and CXCL1 was reversed by ATG5 silencing, suggesting that autophagic activation mediated the effect of DHA on catabolism in chondrocytes (Fig. 3F).

### DHA inhibits NF-κB pathway activated by TNF-α in chondrocytes

**Figure 4:**
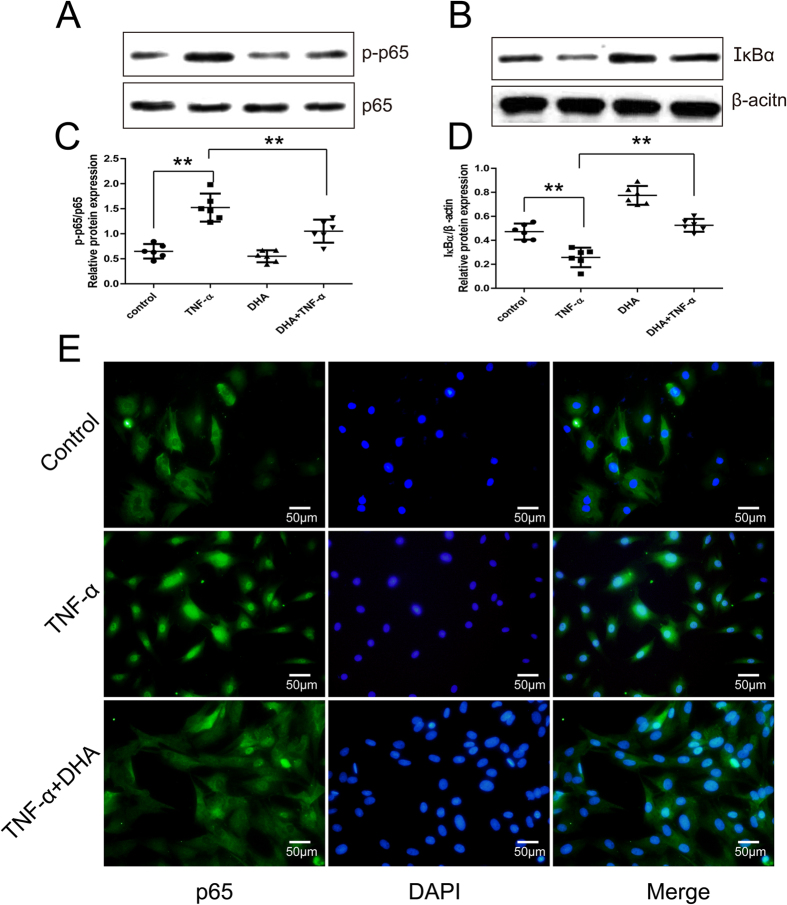
DHA’s effects on the NF-κB pathway in TNF-α-treated chondrocytes for 24 hours. Representative images are shown. (**A**,**C**) Western blotting of p-p65 expression in TNF-α- and/or DHA -treated chondrocytes and the optical density of p-p65/p65 were analyzed. Data represent as mean ± 95% CI, **P < 0.01, n = 6. (**B**,**D**) Western blotting analysis of IκBα expression in TNF-α and/or DHA-treated chondrocytes, and the optical density of IκBα/β-actin was analyzed. Data represent mean ± 95% CI, **P < 0.01, n = 6. (**E**) Immunofluorescence of NF-κB p65 in TNF-α- and/or DHA-treated chondrocytes.

Western blotting of p-p65 and IκBα and immunofluorescence of NF-κB p65 was used to analyze the degree of NF-κB pathway activation. As expected, TNF-α significantly enhanced the levels of p-p65 and decreased the expression of IκBα protein in chondrocytes (Fig. 4A–D). Nuclear translocation of NF-κBp65 in TNF-α-treated chondrocytes for 24 hours (Fig. 4E) suggested TNF-α stimulation of the NF-κB pathway. In contrast, DHA decreased p-p65 expression and induced IκBα accumulation in chondrocytes (Fig. 4A–D). In addition, the p65 protein distribution in the cytoplasm of the DHA- and TNF-α-treated chondrocytes (Fig. 4E) suggested that nuclear translocation of p65 was partially reversed by DHA treatment.

### Involvement of NF-κB pathway in the autophagic activation induced by DHA

**Figure 5:**
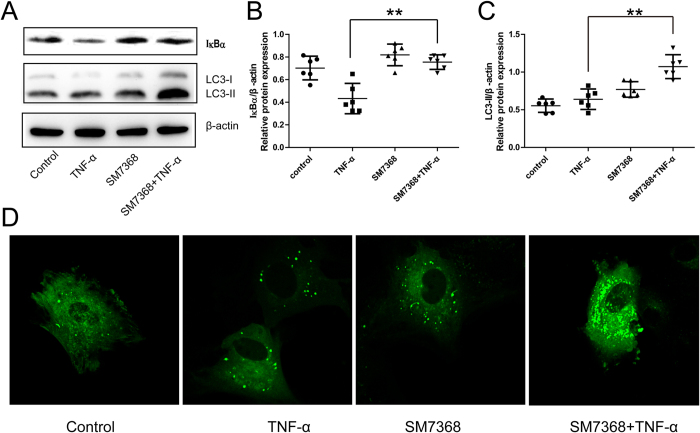
Involvement of the NF-κB pathway in autophagic activation in chondrocytes under inflammatory conditions for 24 hours. (**A**,**B**,**C**) Chondrocytes were treated with the TNF-α and/or NF-κB pathway inhibitor SM7368, and Western blotting analysis of IκBα and LC3 expression was done. The optical density of IκBα/β-actin and LC3-II/β-actin was analyzed. Data represent mean ± 95% CI, **P < 0.01. (**D**) The autophagic level in chondrocytes was analyzed by GFP-LC3 assay.

The inhibition of NF-κB pathway involved in the autophagic activation was investigated using SM7368, an NF-κB inhibitor. IκBα expression was enhanced after SM7368 treatment in TNF-α-treated chondrocytes for 24 hours (Fig. 5A,B), suggesting inhibition of the NF-κB pathway. Importantly, LC3-II levels were increased by SM7368 in TNF-α-induced chondrocytes (Fig. 5A,C). GFP-LC3 analysis showed that the number of green puncta in the cytoplasm was also increased after treatment with SM7368 (Fig. 5D), indicating autophagy activation.

**Figure 6:**
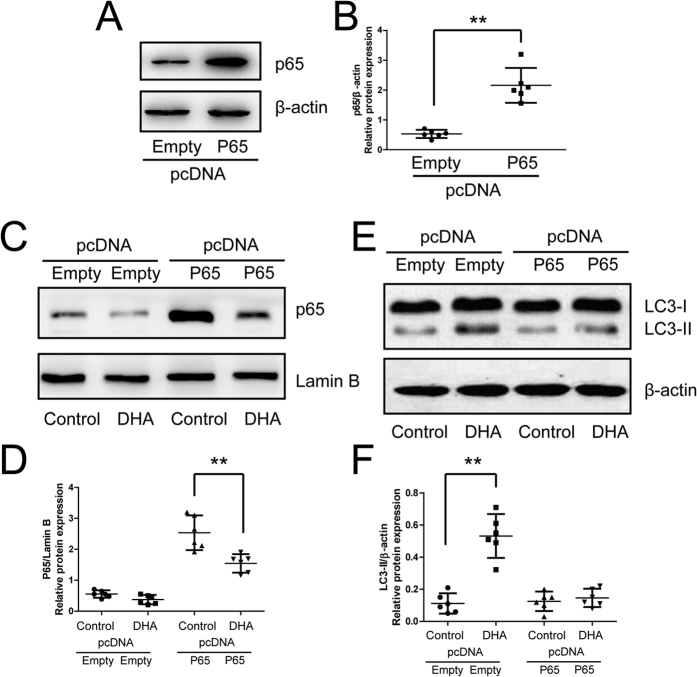
Suppression of autophagic response to DHA by p65 over-expression in chondrocytes. (**A**,**B**) Chondrocytes transfected with p65 plasmid and analyzed by Western blotting of p65 expression 48 hours after transfection. The optical density of p65/β-actin was analyzed. Data represent as mean ± 95% CI, **P < 0.01. (**C**,**D**,**E**,**F**) Chondrocytes transfected with p65 or empty plasmid were treated with DHA for 24 hours, and the expression of nuclear p65 and total LC3-II was examined by Western blotting. The optical density of p65/Lamin B and LC3-II/β-actin was analyzed. Data represent mean ± 95% CI, **P < 0.01.

Further, chondrocytes with p65 over-expression were used to investigate the involvement of NF-κB pathway in autophagic activation (Fig. 6A,B). DHA promoted the expression of LC3-II/β-actin in the chondrocytes transfected with empty plasmid. However, in the chondrocytes transfected with p65 plasmid, the stimulatory role of DHA on LC3-II expression was significantly inhibited (Fig. 6E,F), suggesting the involvement of p65 and NF-κB pathway in the autophagic response to DHA.

## Discussion

In the present study, we demonstrated that 1 μM DHA treatment had no adverse effects on chondrocytes after 24 hours. The increased levels of LC3-II and ATG5 as well as the number of positive autophagosomes demonstrated DHA-activated autophagy, visualized under TEM and confocal microscopy. Treatment with bafilomycin A1 demonstrated the DHA-induced autophagic flux. DHA inhibited the expression of TNF-α-induced catabolic genes. The inhibition was reversed by autophagic inhibition via ATG5-siRNA, suggesting that autophagic activation mediated DHA inhibition of catabolism in chondrocytes. DHA inhibited TNF-α-stimulated NF-κB signaling, confirmed by Western blotting for IκB-α and p-p65, as well as p65 immunofluorescence. Inhibition and over-expression of the NF-κB pathway using SM7368 and p65 plasmid transfection validated the role of NF-κB pathway in DHA-induced autophagy in chondrocytes.

The cytotoxic effects of DHA on several cancer cell types such as pancreatic cancer, glioma l and osteosarcoma have been extensively demonstrated^9^,^22^. However, the dose of DHA used in these studies was in excess of 1 μM, the dose used in the present study. The application of DHA in a variety of human diseases other than malaria may also indirectly reflect its safety^23^. Treatment with 40 μM artesunate (a derivative of artemisinin) after 24 hours failed to reduce the viability of fibroblast-like synoviocytes^24^. Similarly, exposure to 0.5, 1, and 2.5 μM DHA after 48 hours demonstrated no cytotoxicity on bone marrow macrophages in mice^6^. In the present study, exposure to DHA levels 2.5 μM and greater after 48 and 72 hours inhibited cell proliferation, Whereas concentrations of 1 μM DHA or less had no inhibitory effect on chondrocytes.

Due to the regulatory role of multiple signaling pathways, artemisinin has been shown to exert anti-inflammatory and anti-catabolic effects in various disease models^25^. Artemisinin was shown to alleviate neuroinflammation in a model of Alzheimer’s disease^26^. Xu H *et al*. found that artesunate inhibited the expression of TNF-α-induced pro-inflammatory cytokines via suppression of NF-κB pathway in fibroblast-like synoviocytes in human rheumatoid arthritis^24^. Cartilage and bone destruction, as well as catabolic genes, including MMP-2 and -9, decreased after artesunate treatment in collagen-induced arthritis^27^. In our study, catabolic genes, including MMP-3 and -9 and ADAMTS5, and the proinflammatory cytokines, including CCL-2 and-5 and CXCL1, were also inhibited by DHA treatment, suggesting the potential role of DHA in maintaining cellular homeostasis and treating osteoarthritis. However, the MMP13 expression was not altered by the DHA and TNF-α, which was consistent with the results of our previous study involving LPS-treated chondrocytes^15^. Furthermore, MMP13 is a major contributor to cartilage degradation. Therefore, the effect of DHA on catabolism requires to be further investigated.

In this study, the mechanisms involved in DHA effects on catabolism and inflammation in chondrocytes were investigated. Autophagy was found to mediate DHA’s effects in chondrocytes. The stimulatory role of DHA on autophagy was demonstrated by Western blotting analysis of LC3-II and ATG5, GFP-LC3 assay, TEM and autophagic flux assay using bafilomycin A1. However, ATG5-siRNA-induced autophagic inhibition reversed the expression of DHA-inhibited catabolic genes and chemokines. Consistent with our results, autophagy was also activated by DHA in myeloma cancer and glioma cells^20^,^28^. In the HeLa and HCT116 cells, DHA induced autophagy by upregulating the level of stress-regulated protein p8, and the activation of autophagy attenuated the DHA-induced apoptosis adaptively^29^. In the ovarian cancer cells, autophagy was also activated by DHA via the mTOR pathway, although the association between apoptosis and autophagy was not proven^30^. JNK-mediated Beclin 1 expression was demonstrated to be involved in the DHA-induced autophagic activation in human pancreatic cancer cell lines^31^. Autophagy controlling cellular homeostasis has been found to inhibit catabolism and inflammation in cells under stress. In OA cartilage in mice, autophagic activation decreased ADAMTS5 and IL-1β expression as well as inflammation^11^. Reactive oxygen species (ROS) were suggested to be a target of autophagic activity on catabolism and inflammation in chondrocytes^32^. ROS were involved in the TNF-α-induced catabolism, and ROS inhibition could be attributed to removal of aggregation-prone proteins by autophagy.

The NF-κB family comprises of a group of transcription factors, which are activated by pro-inflammatory factors, chemokines, and cytokines in OA^33^. In the present study, p65 was mainly expressed in the cytoplasm in the normal chondrocyte. However, TNF-α increased p65 expression and stimulated its nuclear translocation by inducing degradation of IκB-α which combined with p65 in the cytoplasm, reflecting the activation of the NF-κB pathway in chondrocytes. Consistent with results found in other cancer cells, fibroblast-like synoviocytes, and osteoclasts, DHA inhibited p65 nuclear translocation and reduced IκB-α degradation, suggesting that DHA inhibites the NF-κB pathway by modulating the IκB-α protein^6^,^20^,^24^. Although the inhibitory role of DHA on NF-κB has been extensively studied, the association between NF-κB and autophagy in chondrocytes is unclear. In our study, the NF-κB inhibitor SM7368 was shown to increase LC3-II levels and autophagosomal numbers in TNF-α-treated chondrocytes. Furthermore, p65 over-expression via transfection of p65 plasmid inhibited the autophagic response to DHA in chondrocytes, directly demonstrating the role of NF-κB in DHA-induced autophagy. In nuclear pulposus cells, NF-κB and c-Jun N-terminal kinase (JNK) were suggested to be involved in autophagic activation^19^. In porcine primary granulosa cells, Gao *et al*. found that NF-κB activated autophagy via activation of the JNK pathway^34^. The JNK pathway might be associated with the up-regulation of autophagy in cancer cells. However, the detailed mechanisms underlying NF-κB inhibitory effects on autophagic activation require further investigation.

In conclusion, the present study demonstrated that exposure to 1 μM DHA has no cytotoxic effects on chondrocytes. DHA induces autophagic activation and inhibits the expression of TNF-α-induced catabolic genes and chemokines. An important finding relates to the reversal of DHA-induced inhibition on catabolism and inflammation by autophagy inhibition via ATG5siRNA transfection. DHA suppresses the activation of the NF-κB pathway stimulated by TNF-α. Finally, the NF-κB pathway inhibitor SM7368 activates autophagy in TNF-α-treated chondrocytes. However, p65 over-expression reduced the autophagic response to DHA. Our findings further our understanding of DHA-induced autophagic activation via NF-κB pathway.

## Materials and Methods

All the experimental protocols for animals in this study were approved by the Fudan University Animal Care and Use Committee (Shanghai, China). All the following methods were carried out in accordance with the relevant guidelines and regulations.

### Reagents and antibodies

CCK-8 (Cell Counting Kit-8) reagent was purchased from Dojindo (Tokyo, Japan). The autophagy protein 5 (ATG5), microtubule-associated protein light chain 3 (LC3), p-p65, p65, and IκBα primary antibodies for Western blotting were purchased from Cell Signaling Technology (Massachusetts, USA), and the other reagents used for Western blotting, including the horseradish peroxidase-conjugated secondary antibodies, radioimmunoprecipitation assay (RIPA), polyvinylidene difluoride (PVDF) membranes, and sodium dodecyl sulfate-polyacrylamide gel electrophoresis (SDS-PAGE) were obtained from Beyotime (Shanghai, China). The p65 primary antibody for immunofluorescence was supplied by Abcam (Cambridge, UK). Adenovirus expressing green fluorescent protein–microtubule-associated protein light chain 3(GFP–LC3) was purchased from Genomeditech (Shanghai, China). Acridine orange (AO), TNF-α, DHA, and SM7368 were purchased from Sigma-Aldrich (St. Louis, MO, USA).TRIzol and the SYBR Premix Ex Taq mixture were obtained from Takara (Takara Bio, Otsu, Japan).

### Rat chondrocyte cultures

The chondrocyte extraction procedure and culture methods have been described briefly in a previous paper^15^. After euthanasia with high-dose 10% (w/v) chloral hydrate, knee articular cartilages from five 250‒300 g male SD rats were separated and collected under sterile conditions. Primary cell culture was repeated six times, with five rats used each time. The cartilage tissues were transferred to a clean bench and cut into pieces using a micro-scissor. The tissues were digested with 0.25% Trypsin-EDTA for 30 min and collagenase II for 4 h and then filtered. After rinsing, the chondrocytes were cultured with Dulbecco’s modified Eagle’s medium (DMEM) with high-dose (4.5 g/L) glucose, 10% FBS, and 1% penicillin/streptomycin at 37 °C with 5% CO_2_. Second passage chondrocytes were used in the experiments to eliminate the influence of de-differentiation on experimental results.

### Experimental design

DHA cytotoxicity was assessed first, followed by an investigation of autophagic levels after 1 μM DHA treatment for 24 hours. The H^+^-ATPase inhibitor bafilomycin A1 (0.1 μM) was used to measure the role of 1 μM DHA or 1 μM DHA combined with 50 ng/mL TNF-α in autophagic flux. Autophagic involvement in the inhibitory role of 1 μM DHA on catabolic genes was then analyzed by ATG small interfering RNA (siRNA). Finally, the NF-κB pathway involved in autophagic activation was investigated. When cells were treated with 50 ng/mL TNF-α and 1 μM DHA or 10 μM SM7368, DHA and SM7368 were always added to the medium 1 h prior to TNF-α addition. In all the experiments, chondrocytes were treated with TNF-α for 24 hours. During performing autophagic flux assay, 0.1 μM bafilomycin A1 was added to the medium 1 h prior to 1 μM DHA or 1 μM DHA combined with 50 ng/mL TNF-α, followed by incubation for 24 hours together.

### CCK-8 test

Chondrocytes (5 × 10^3^ in each well) were cultured in a 96-well plate overnight, followed by treatment with 0‒10 μM DHA for 24, 48, and 72 hours. After treatment, 10 μL CCK-8 and 100 μL medium were added to the wells and the cells were cultured at 37 °C for 2 hours. The absorbance was measured in a microplate reader at 450 nm.

### Western blotting

After treatments as described in the experimental design, chondrocytes in 10 cm dishes were washed with cold phosphate-buffered saline (PBS) and incubated with RIPA containing 1 mM phenylmethylsulfonyl fluoride, followed by cell scraping and centrifugation. The nuclear protein was isolated as recommended by the manufacturer (Beyotime, Shanghai, China). After measuring protein concentration with the bicinchoninic acid (BCA) assay, 40 μg total protein containing loading buffer was separated by SDS-PAGE and transferred to PVDF membranes. The membranes were immersed in 5% nonfat milk for 2 h to block non-specific antigen and then incubated with primary antibodies (LC3, 1:1000; ATG5, 1:800; p-p65, 1:500; p65, 1:500; IκBα, 1:500) overnight at 4 °C. After incubation with secondary antibodies (1:2000), an enhanced chemiluminescence detection system (PerkinElmer, USA) with an ECL reagent was used to expose proteins on the membrane. A semi-quantitative analysis of protein bands was performed using AlphaEaseFC 4.0 software.

### GFP-LC3 analysis

In order to observe cells under a confocal microscope, 5 × 10^4^ chondrocytes were cultured overnight in 3.5 cm glass-bottom dishes in 2 mL DMEM with 10% FBS. Chondrocytes were then incubated for 2 h with GFP-LC3 adenovirus at a multiplicity of infection of 100 in 1 mL DMEM without FBS for 2 h, according to the manufacturer’s instructions. After washing with PBS, 2 mL DMEM with 10% FBS was used to incubate chondrocytes overnight to eliminate the effect of starvation on autophagy. The transfection efficiency was observed under a fluorescence microscopy, and treatments were then administered. Finally, the confocal microscopy (Leica TCS SP8, Germany) was used to visualize the autophagosomes and autolysosomes in the chondrocytes.

### Transmission electron microscopy (TEM)

Chondrocytes cultured in 10 cm dishes were treated with TNF-α and/or DHA for 24 hours, and the cells were removed by hand scraping. After centrifugation, chondrocytes were fixed with 2.5% glutaraldehyde overnight and post-fixed with 1% osmium tetroxide for 2 h at 4 °C. After staining with 2% uranyl acetate, chondrocyte pellets were dehydrated with an acetone series and then embedded in Epon 812. After semi-thin sectioning, chondrocytes were stained with toluidine blue and observed under the microscope. Finally, ultra-thin sections were performed according to the above observations. Cellular ultra-structures were visualized under a transmission electron microscope (Hitachi, Japan).

### AO staining

AO staining was used to detect acidic vesicular organelles in chondrocytes. AO dye was diluted with the DMEM without FBS to 5 μg/mL. After different treatments, chondrocytes were rinsed with PBS and incubated with 5 μg/mL for 15 min at 37 °C. Chondrocytes were then observed under the fluorescence microscope.

### Real-time PCR

Chondrocytes (1 × 10^5^ in each well) cultured in a six-well plate were washed with cold PBS and incubated with TRIzol reagent to extract total RNA. cDNA was synthesized using 1 mg total RNA with a cDNA synthesis kit (MBI Fermantas, Germany). The primers of MMP3, -9 and-13, ADAMTS4 and-5, CCL-2 and-5, and CXCL1 are shown in Table 1. tive mRNA levels of those genes were calculated using the 2^−ΔΔCt^ method. Twenty microliters of reaction volume was prepared before amplification, including 2 μL of twofold diluted cDNA, 10 μL 2 × SYBR Premix Ex Taq mixture (Takara, Japan), 0.2 μM each primer, and sterile distilled water. The amplification reaction was performed on a Mastercycler^®^ ep realplex platform (Eppendorf, Hamburg, Germany). After amplification, the cycle threshold (Ct) values were obtained and normalized to the value of the housekeeping gene glyceraldehyde 3-phosphate dehydrogenase. The relative mRNA levels of those genes were calculated using the 2^−ΔΔCt^ method.

### Immunofluorescence

Chondrocytes (5 × 10^4^ in each well) in a 24-well plate were treated as described in the design and fixed with 4% paraformaldehyde for 10 min at 37 °C. After washing with PBS and incubation with 0.2% Triton X-100 for 15 min, chondrocytes were incubated with 5% goat serum for 30 min and primary p65 antibody (1:150) overnight at 4 °C followed by incubation with Alexa Fluor488-conjugated secondary antibody. 4′,6-Diamidino-2-phenylindole (Beyotime, China) was used to counterstain the nuclei. Chondrocytes were visualized under a fluorescence microscope.

### Atg5 siRNA and p65 plasmid transfection

Based on our previous study^35^, the sequences of ATG5 siRNAs for ATG5 were designed and synthesized by GenePharma (Shanghai, China). The detailed sequences are shown in Table 2. Chondrocytes were cultured in a six-well tissue culture plate (2 × 10^5^ cells in each well). Lipofectamine2000 (Invitrogen) was used to deliver siRNA into chondrocytes according to the manufacturer’s instructions. Forty-eight hours after transfection, the infected chondrocytes were treated as described in the experimental design. A non-specific, non-targeting siRNA (scrambled) was used as a control. 48 hours after transfection, the inhibitory efficiency of specific silencing was examined using the Western blotting.

In order to over express p65, the p65 complete ORF sequences were cloned into pcDNA3.1(+) expression vector (Invitrogen, Carlsbad, CA). All the vectors have been verified via sequencing by Invitrogen Inc (Shanghai, China). The transfection method was similar to the way by which ATG5 siRNA was delivered into chondrocytes. 48 hours after transfection, Western blotting of p65 was performed to analyze the transfection efficiency.

### Statistical analysis

SPSS 20 statistical software (SPSS Inc., Chicago, IL, USA) was used to do the statistical analysis. First, the normal distribution of data was verified. Analysis of variance was used to detect the differences between these groups. A Tukey test analysis was used to explore the differences between the two groups if necessary. All the experiments were repeated six times using different chondrocytes collected from five rats each time, and the primary cell culture was performed six times. The differences were considered to be statistically significant at P < 0.05.

## Additional Information

**How to cite this article**: Jiang, L.-B. *et al*. Dihydroartemisinin inhibits catabolism in rat chondrocytes by activating autophagy via inhibition of the NF-kB pathway. *Sci. Rep.* **6**, 38979; doi: 10.1038/srep38979 (2016).

**Publisher's note:** Springer Nature remains neutral with regard to jurisdictional claims in published maps and institutional affiliations.

## Figures and Tables

**Table 1:** Primer sequences for MMPs, ADAMTSs, chemokines, and GAPDH.

Gene		Sequences (5′–3′)	Product size (bp)	Accession no.
MMP3	forward	ATGAACGATGGACAGATGA	19	NM_133523.3
		TGAGAGAGATGGAAACGG	18	
MMP9	forward	GTCTTCCCCTTCGTCTTC	18	NM_031055.1
		AAACCCCACTTCTTGTCAG	19	
MMP13	forward	ATGAAACCTGGACAAGCA	18	NM_133530.1
		GGACCATAGAGAGACTGGATT	21	
ADAMTS4	forward	CGTGGTGTGTGTGTGTGT	18	NM_023959.1
		AGAGGAAAGTAGGGCAGGT	19	
ADAMTS5	forward	GTGTGTGGAGGGGATAACT	19	NM_198761.1
		TCTGGTCTTTGGCTTTGA	18	
CCL2	forward	TGCTGACCCCAATAAGGAATG	21	NM_031530.1
		TGCTGACCCCAATAAGGAATG	22	
CCL5	forward	GACACCACTCCCTGCTGCTT	20	NM_031116.3
		ACACTTGGCGGTTCCTTCG	19	
CXCL1	forward	GAAGATAGATTGCACCGATG	20	NM_030845.1
		CATAGCCTCTCACACATTTC	20	
GAPDH	forward	CAACGGGAAACCCATCACCA	20	NM_017008.3
		ACGCCAGTAGACTCCACGACAT	22	

GAPDH = glyceraldehyde phosphate dehydrogenase; MMP = matrix metalloproteinase; ADAMTS = a disintegrin and metalloproteinase with thrombospondin motifs; CCL = CC chemokine ligand; CXCL = CXC chemokines ligand.

**Table 2:** Small interfering RNA (siRNA) sequences.

Primer	Direction	Sequence 5′–3′
*Atg5*	Sense	GGCCUUUCAUUCAGAAGCUTT
	Antisense	AGCUUCUGAAUGAAAGGCCTT
Negative control	Sense	UUCUCCGAACGUGUCACGUTT
	Antisense	ACGUGACACGUUCGGAGAATT
